# Supplementary material for: In situ architecture of neuronal α-Synuclein inclusions
Source: Nat Commun. 2021 Apr 14;12:2110. doi: 10.1038/s41467-021-22108-0 (PMC8046968; doi:10.1038/s41467-021-22108-0)
Supplement: Supplementary file 1 — Supplementary Information [file 41467_2021_22108_MOESM1_ESM.pdf]

In situ architecture of neuronal α-Synuclein inclusions

V.A. Trinkaus et al.

Supplementary Files

Contents

Supplementary Table 1

Supplementary Table 2

Supplementary Fig. 1

Supplementary Fig. 2

Supplementary Fig. 3

Supplementary Fig. 4

Supplementary Fig. 5

Supplementary Fig. 6

Supplementary Fig. 7

Supplementary Methods

Supplementary Table 1 | Statistics of cryo-ET experiments on mouse neurons.

Condition	Experiments	Analyzed tomograms	Analyzed filaments	Analyzed membrane area (µm²)
GFP-α-syn + PFFs	3	6	1592	4.11
Endogenous α-syn + PFFs	3	4	220	2.87
GFP-α-syn + MSA	3	5	721	3.70
Untransduced - PFFs	2	5	-	3.67

Neurons were either transduced with GFP-α-syn and seeded with PFFs ("GFP-α-syn + PFFs"), transduced with p62-RFP and seeded with PFFs ("Endogenous α-syn + PFFs"), transduced with GFP-α-syn and seeded with aggregates derived from a MSA patient brain ("GFP-α-syn + MSA"), or untransduced and unseeded as control ("Untransduced - PFFs"). The column "Experiments" lists biologically independent replicates. "Analyzed filaments" includes all filaments analyzed in Fig. 2d, e, Fig. 4 and Supplementary Fig. 7d. "Analyzed membrane area" includes all membranes analyzed in Fig. 4 and Supplementary Fig. 7e.

Supplementary Table 2 | List of primers.

Primer name	Sequence
Forward primer pFhSynW2 GFP-synA53T-Flag	GCA GTC GAG AGG ATC CCG GGC CCA CCA TGG TGA GCA AGG GCG AG
Reverse primer pFhSynW2 GFP-synA53T-Flag	CCG CTC TAG AGC TAG CTT ATT TAT CGT CGT CAT CCT TGT AAT CGG CTT CAG GTT CGT AGT CTT GAT AC
Forward primer pFhSynW2 Flag-GFP	GAG CGC AGT CGA GAG GAT CCC CCA CCA TGG ATT ACA AGG ATG ACG ACG ATA AGC CCG GGA TGG TGA GCA AGG GCG AG
Reverse primer pFhSynW2 Flag-GFP	GCT TGA TAT CGA ATT CTT ACT TGT ACA GCT CGT CCA TGC

Primers used for cloning the pFhSynW2 GFP-synA53T-Flag and pFhSynW2 Flag-GFP constructs.

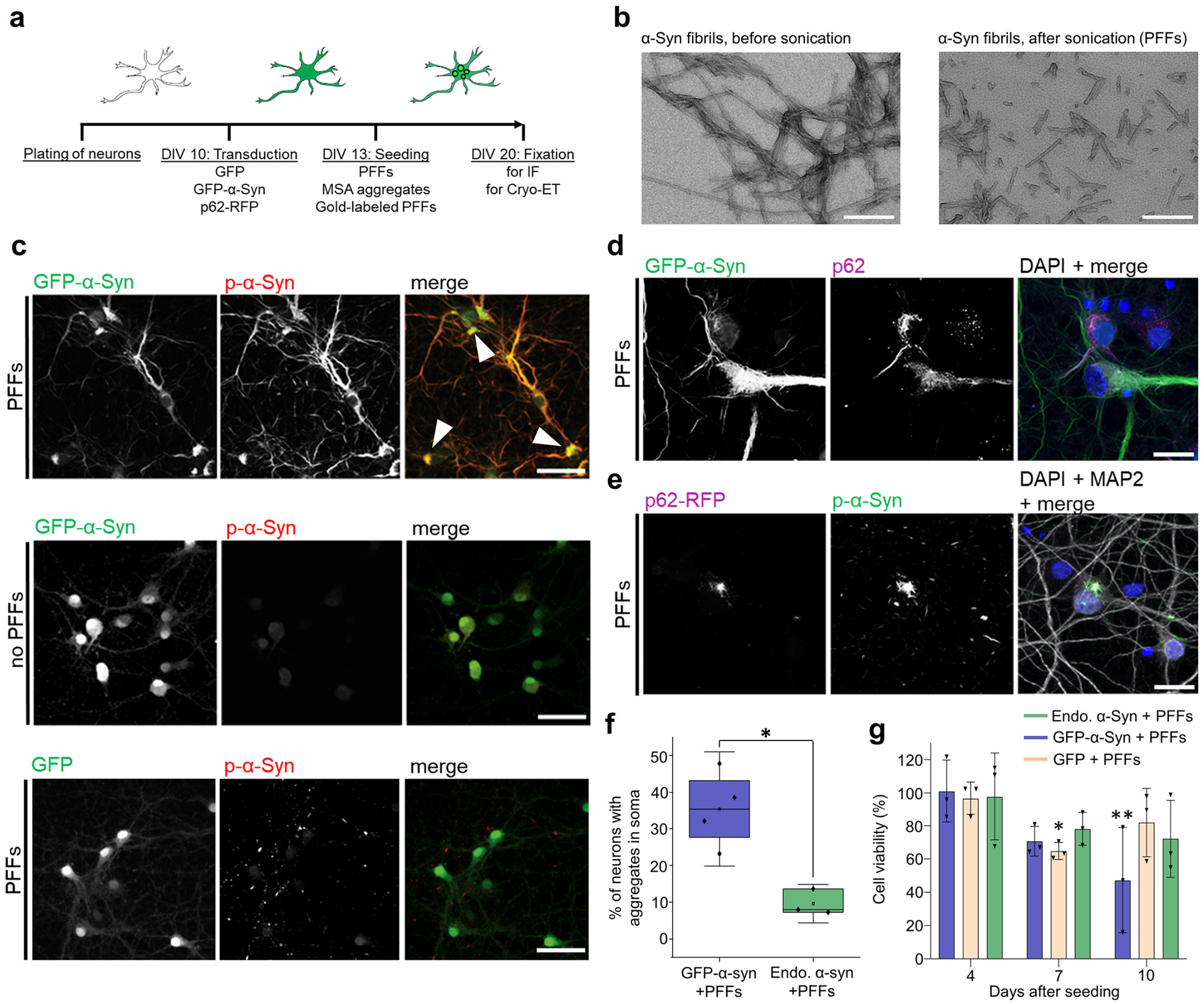

Supplementary Fig. 1 | Seeding of α-Syn aggregates in neurons. a, Schematic of the seeding of α-Syn aggregates in primary neurons. Primary mouse neurons were transduced at day *in vitro* (DIV) 10 with GFP, GFP-α-Syn or p62-RFP. Seeds (PFFs or MSA brain-derived) were applied at DIV 13, and α-Syn inclusions were studied at DIV 20 by light microscopy or cryo-ET upon chemical or cryo-fixation, respectively. For light microscopy imaging, GFP signal was enhanced by staining with an antibody against GFP. **b**, Negative stain images of α-Syn fibrils before (left) and after (right) sonication. Sonicated seeds were used for all seeding experiments. Scale bars: 250 nm. Two biologically independent experiments were performed. **c**, Immunofluorescence imaging of α-Syn aggregates, as detected by an antibody against phosphorylated α-Syn Ser129 (p-α-Syn). Top: aggregate formation (arrowheads) upon seeding cells expressing GFP-α-Syn with exogenous PFFs. Middle: no aggregate formation in cells expressing GFP-α-Syn in the

absence of PFFs. Bottom: PFFs seed smaller aggregates in cells with endogenous α-Syn levels that express GFP only as control (see Supplementary Fig. 1f for quantification). Scale bars: 50 µm. Two biologically independent experiments were performed. **d**, Immunofluorescence imaging of GFP-α-Syn aggregates detected by an antibody against p62. The merged image shows a superposition of the GFP-α-Syn (green), p62 (magenta) and DAPI (blue) channels. An arrowhead indicates the colocalization of GFP-α-Syn and p62. Scale bar: 20 µm. Two biologically independent experiments were performed. **e**, Immunofluorescence imaging of endogenous α-Syn aggregates positive for p-α-Syn colocalizing with p62-RFP. The merged image shows a superposition of the p62-RFP (magenta), phospho-α-Syn (green), the neuronal marker MAP2 (gray) and DAPI (blue) channels. Scale bar: 20 µm. Two biologically independent experiments were performed. **f**, Quantification of the percentage of neurons with aggregates in the soma upon treatment with PFFs of cells transduced with GFP-α-Syn (blue) or untransduced (green; endogenous α-Syn). The horizontal lines of each box represent 75% (top), 50% (middle) and 25% (bottom) of the values, and a black square the average value. Whiskers represent 1.5x standard deviation and black diamonds the individual data points. * indicates $p = 0.011$ by two-tailed unpaired t-test with Welch's correction, $n = 4$ (GFP-α-Syn + PFFs) and 3 (endogenous α-Syn + PFFs) biologically independent experiments. **g**, Quantification of neuronal viability upon seeding with PFFs for cells expressing endogenous α-Syn (Endo. α-Syn + PFFs), or transduced with GFP-α-Syn (GFP-α-Syn + PFFs) or with GFP only (GFP + PFFs) relative to untransduced and unseeded control cells. Bars represent average values, the error bars the standard deviation and black triangles the individual data points. *and ** respectively indicate $p = 0.04$ and $p = 0.002$ by two-way ANOVA and Dunnett's multiple comparison test, $n = 3$ biologically independent experiments for all conditions. Representative images are shown in **b**, **c**, **d**. Source data for **f**, **g** are provided as a Source Data file.

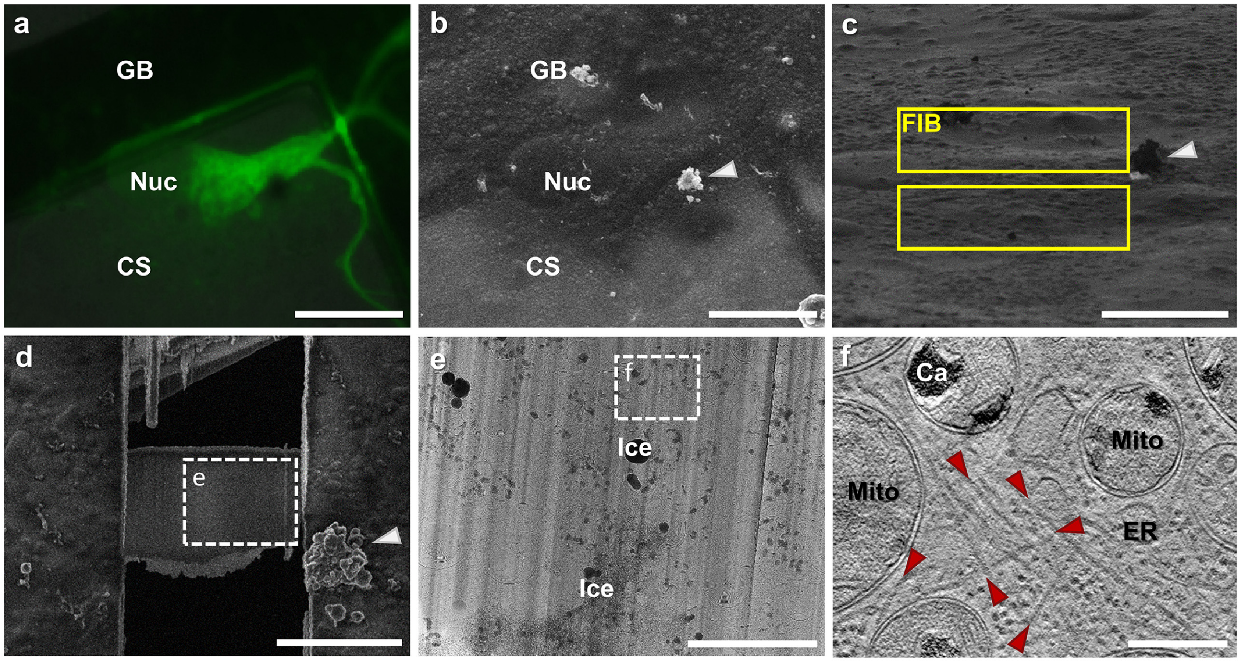

Supplementary Fig. 2 | Cryo-ET workflow. a, Cryo-light microscopy imaging of GFP fluorescence in a primary neuron grown on the carbon support (CS) of an EM grid. The cell was transduced with GFP-α-Syn at DIV 10 and aggregate formation was seeded at DIV 13. The grid was vitrified at DIV 20. GB: grid bar, Nuc: nucleus. Scale bar: 25 µm. **b**, Correlative scanning electron microscopy imaging of the same cell within the cryo-FIB instrument upon coordinate transformation. A white arrowhead marks a piece of ice crystal contamination that can also be found in panels **c** and **d** as visual reference. Scale bar: 25 µm. **c**, FIB-induced secondary electron image of the same cell. Yellow boxes indicate the regions to be milled away by the FIB during lamella preparation. Scale bar: 15 µm. **d**, Scanning electron microscopy imaging of the same cell upon preparation of a 150 nm-thick electron transparent lamella. The white rectangle marks the region of the lamella shown in **e**. Scale bar: 10 µm. **e**, Low magnification transmission electron microscopy image of the area of the lamella marked in **d**. Ice: ice crystal contamination on the lamella surface. The white rectangle marks the region shown in **f**. Scale bar: 3 µm. **f**, A tomographic slice (thickness 1.4 nm) recorded in the area indicated in **e**. Ca: mitochondrial calcium stores, ER: endoplasmic reticulum, Mito: mitochondrion. Red arrowheads indicate α-Syn fibrils. Scale bar: 300 nm. The number of tomograms and biologically independent cryo-ET experiments is listed in Supplementary Table 1. Representative images are shown for all panels.

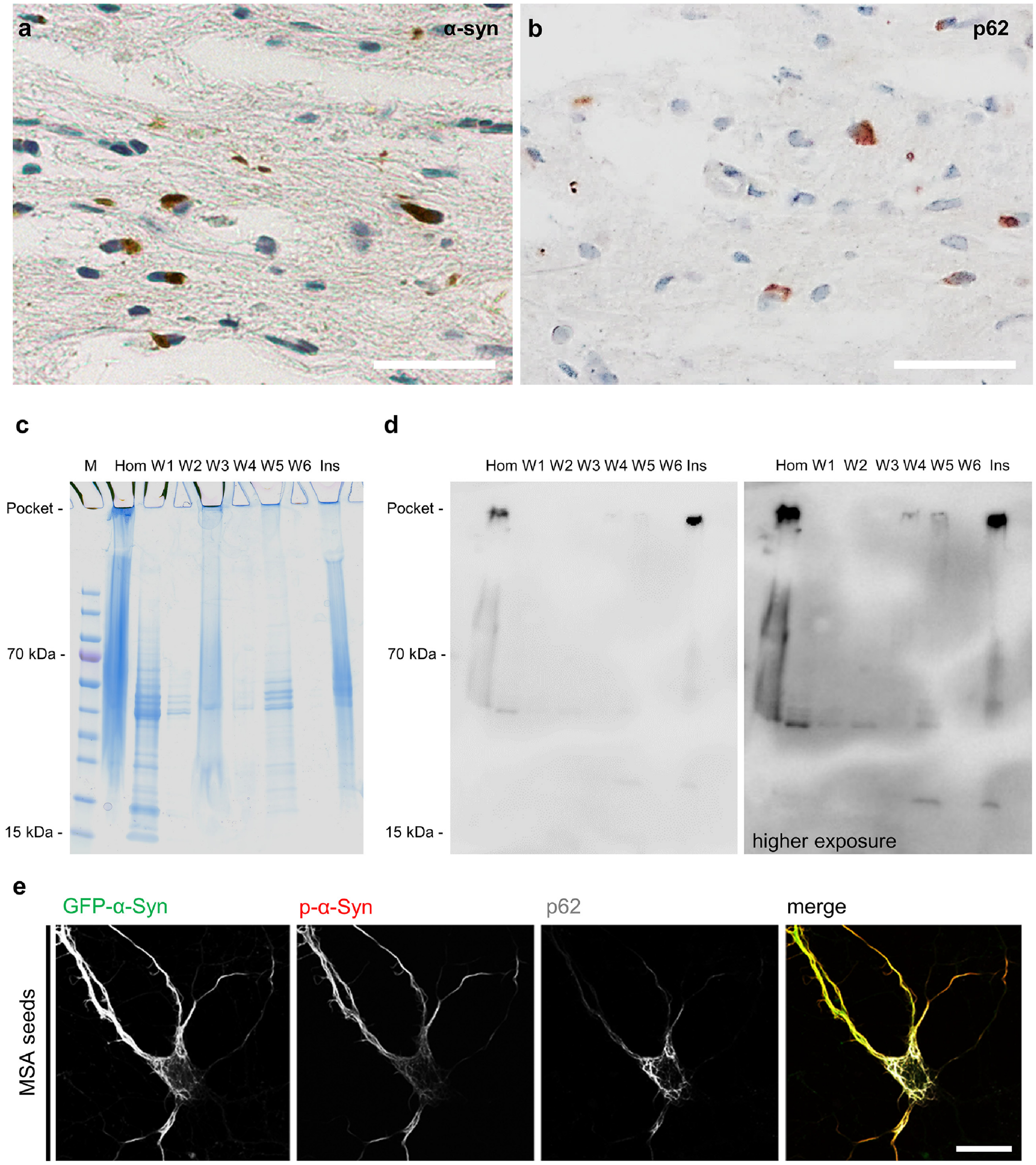

Supplementary Fig. 3 | Purification of α-Syn aggregates from MSA patient brain.

a, **b,** Immunohistochemistry staining showing cytoplasmic inclusions (brown) positive for α-Syn (**a**) and p62 (**b**) in the basilar part of the pons of the brain of an MSA patient. Nuclei are stained in blue. Aggregates for seeding neurons for cryo-ET imaging were purified from the same region

(**c**, **d**). Scale bars: 50 µm. Experiment was performed once. **c**, **d**, Purification of α-Syn aggregates from the MSA patient brain shown in **a**, **b**. Coomassie staining (**c**) and anti-phospho-α-Syn western blot (**d**) of SDS PAGE gels loaded with brain homogenate (Hom), washing fractions (W1-6) and the final sarkosyl-insoluble fraction (Ins) at low (left) and high (right) exposure levels. M: molecular weight marker. Note the aggregated material in the stacking gel. For gel source images, see Supplementary Fig. 2. Experiment was performed once. **e**, Immunofluorescence images of a GFP-α-Syn-expressing neuron seeded with the sarkosyl-insoluble fraction from MSA patient brain, showing aggregates positive for phospho-α-Syn and p62. GFP signal was enhanced by staining with an antibody against GFP. The merged image shows a superposition of the GFP-α-Syn (green), phospho-α-Syn (red) and p62 (gray) channels. Scale bar: 20 µm. Two biologically independent experiments were performed. Representative images are shown for all panels. Source data for **c**, **d** are provided as a Source Data file.

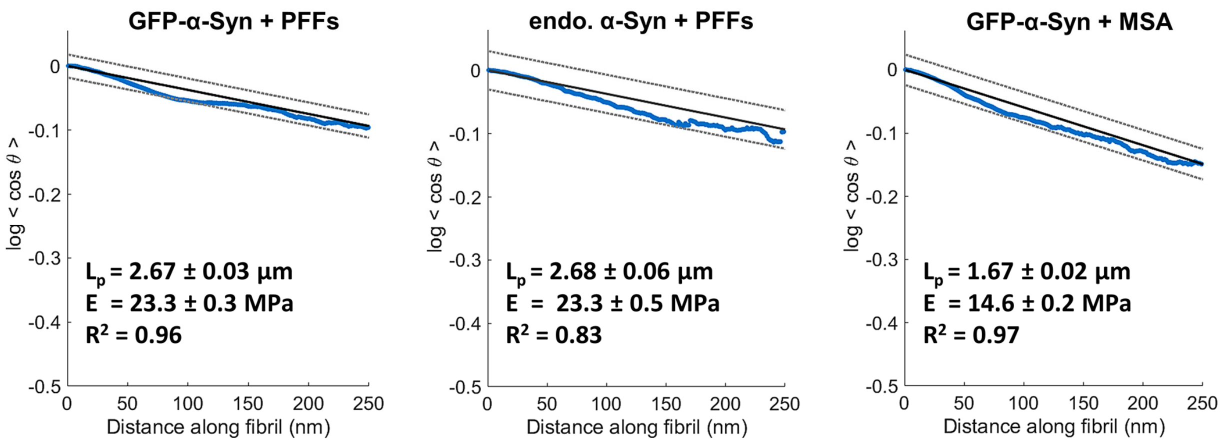

Supplementary Fig. 4 | Persistence length of α-Syn fibrils. Linear fit of the total persistence length for all fibrils analyzed. n = 1295 (GFP-α-Syn + PFFs), 220 (endogenous α-Syn + PFFs) and 721 (GFP-α-Syn + MSA) fibrils in total over two (GFP-α-Syn + PFFs) or three (endogenous α-Syn + PFFs and GFP-α-Syn + MSA) biologically independent experiments. The blue curves represent the original data. 95% confidence interval (dotted lines) and the values of the persistence length (L_p), Young's modulus (E) and coefficients of determination (R^2) are indicated. Note that the values are almost identical for GFP-α-Syn and endogenous α-Syn seeded with PFFs, but lower for GFP-α-Syn seeded with MSA patient aggregates. Source data are provided as a Source Data file.

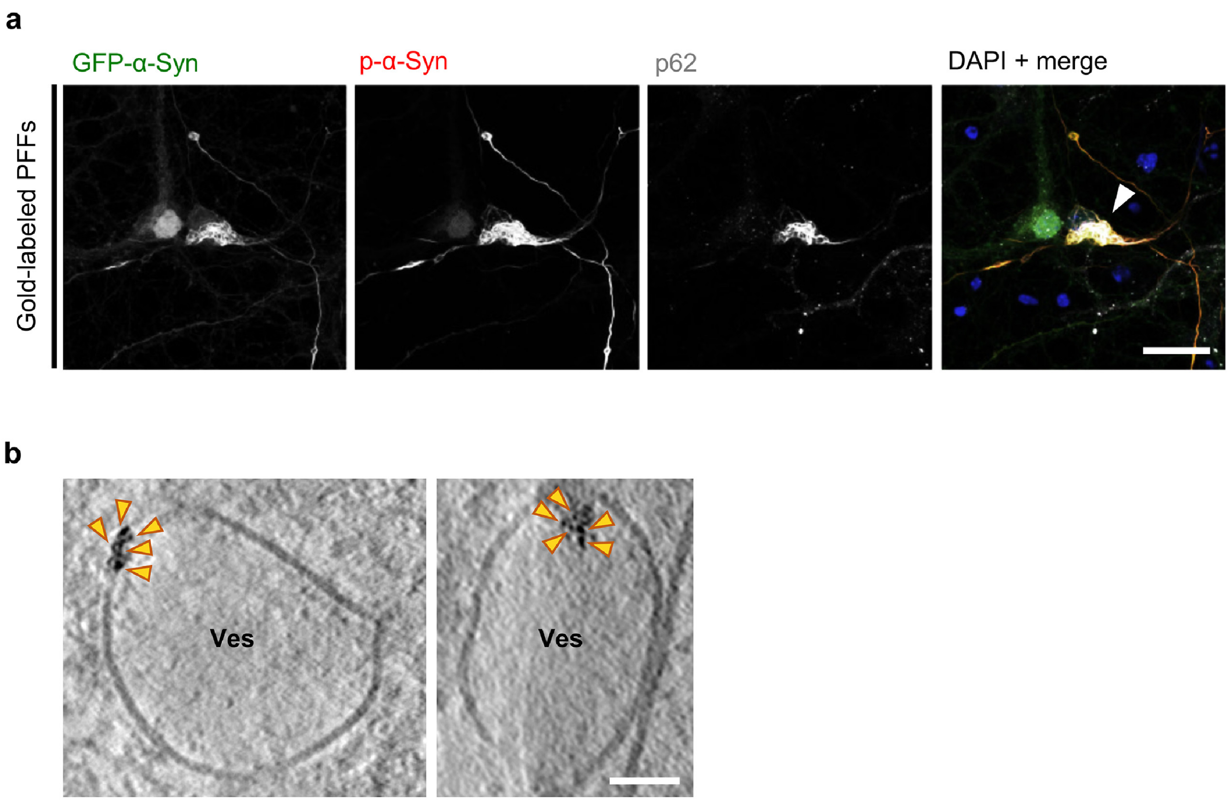

Supplementary Fig. 5 | Seeding of α-Syn aggregates in neurons by gold-labeled PFFs. a, Immunofluorescence images of a GFP-α-Syn-expressing neuron seeded with gold-labeled PFFs. The cells develop α-Syn aggregates, as detected by antibodies against phosphorylated α-Syn Ser129 and p62. GFP signal was enhanced by staining with an antibody against GFP. The merged image shows a superposition of the GFP-α-Syn (green), phospho-α-Syn (red), p62 (gray) and DAPI (blue) channels. An arrowhead indicates the GFP-α-Syn aggregates. Scale bar: 20 µm. **b**, Tomographic slices (thickness 1.4 nm) showing accumulations of gold particles (orange arrowheads) at the membrane (left) or in the lumen (right) of intracellular vesicles. Ves: vesicles. Scale bar: 50 nm. Two biologically independent experiments were performed in all cases. Representative images are shown for all panels.

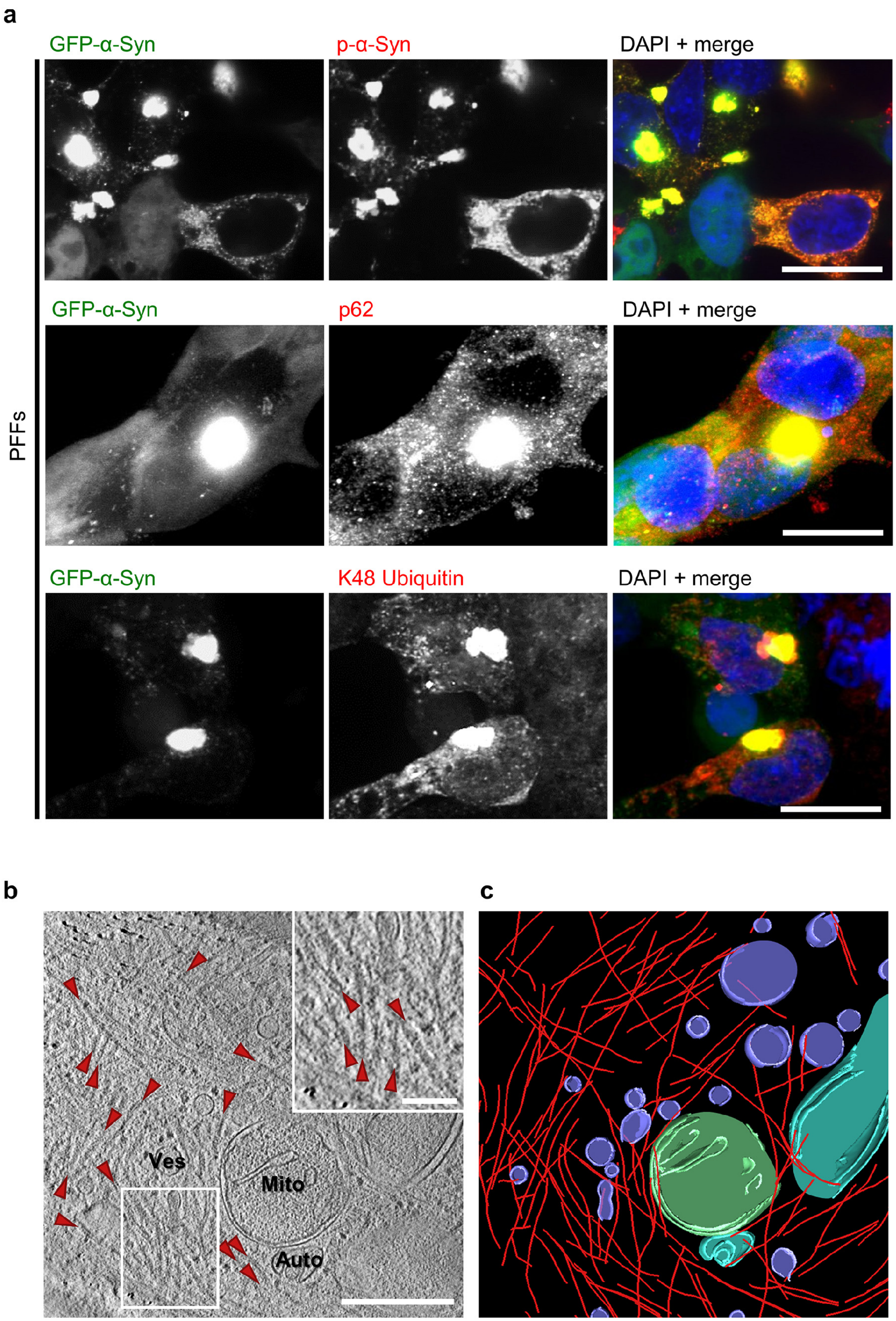
a
GFP-α-Syn
p-α-Syn
DAPI + merge
GFP-α-Syn
p62
DAPI + merge
GFP-α-Syn
K48 Ubiquitin
DAPI + merge
PFFs
b
Ves
Mito
Auto
c

Supplementary Fig. 6 | α-Syn aggregates in SH-SY5Y cells. a, Immunofluorescence images of SH-SY5Y cells stably expressing GFP-α-Syn and seeded with PFFs. The cells develop α-Syn inclusions, as detected by antibodies against phosphorylated α-Syn Ser129 (top), p62 (middle) and K48-linked ubiquitin (bottom). The merged images show a superposition of the respective green and red channels plus DAPI (blue). Scale bars: 15 µm. **b**, A tomographic slice (thickness 1.8 nm) of an inclusion seeded by PFFs in a SH-SY5Y cell expressing GFP-α-Syn. Auto: autophagosome; Mito: mitochondrion; Ves: vesicles. Fibrils are marked by red arrowheads. Scale bars: 350 nm (main panel) and 100 nm (inset). **c**, 3D rendering of the tomogram depicted in **b** showing α-Syn fibrils (red), autophagosomes (cyan), mitochondria (green) and various vesicles (purple). Three biologically independent experiments were performed in all cases. Representative images are shown for all panels. Source data are provided as a Source Data file.

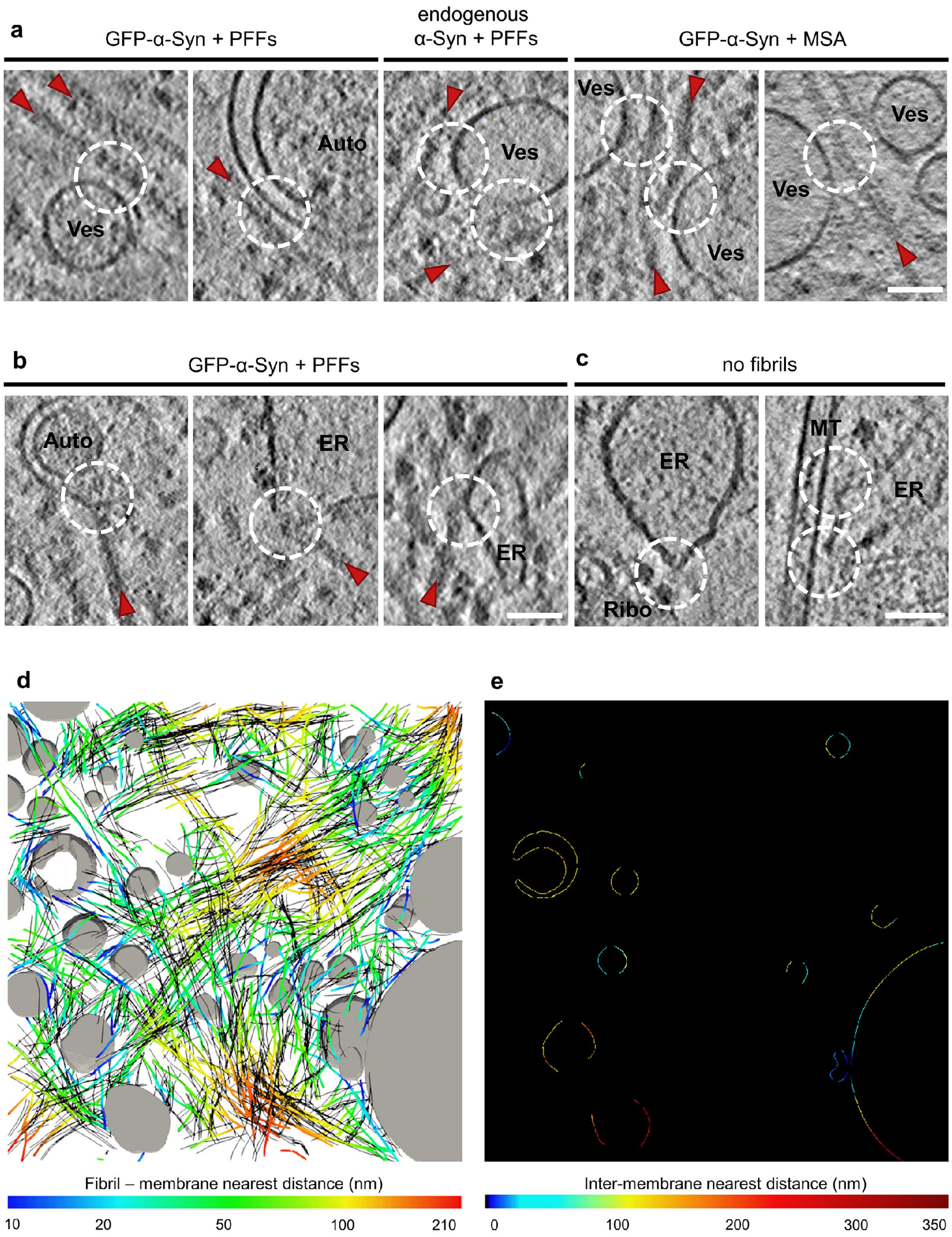

a
GFP-α-Syn + PFFs
endogenous α-Syn + PFFs
GFP-α-Syn + MSA
Ves
Auto
Ves
Ves
Ves
Ves
Ves
b
GFP-α-Syn + PFFs
Auto
ER
ER
c
no fibrils
ER
Ribo
MT
ER
d
e
Fibril – membrane nearest distance (nm)
10
20
50
100
210
Inter-membrane nearest distance (nm)
0
100
200
300
350

Supplementary Fig. 7 | Proximity of α-Syn fibrils and cellular membranes. a, Gallery of tomographic slices showing close proximity events (dashed white circles) between α-Syn fibrils (red arrowheads) and different cellular membranes with no apparent interactions. Auto: autophagosome, Ves: vesicles. Tomographic slices are 1.8 nm (GFP-α-Syn + PFFs) or 1.4 nm (endogenous α-Syn + PFFs and GFP-α-Syn + MSA) thick. Scale bar: 50 nm. **b,** Gallery of tomographic slices (thickness 1.8 nm) showing apparent contacts between α-Syn fibrils and different cellular membranes at sites of high membrane curvature (dashed white circles), within inclusions seeded by PFFs in neurons expressing GFP-α-Syn. ER: endoplasmic reticulum. Scale bar: 50 nm. **c,** Tomographic slices showing sites of high membrane curvature (dashed white circles) in the absence of α-Syn fibrils in neurons expressing p62-RFP and seeded with PFFs. MT: microtubule; Ribo: ribosome. Tomographic slices are 1.4 nm thick. Scale bar: 60 nm. **d**, 3D rendering shown in Fig. 1d and Fig. 4a with α-Syn fibrils color-coded according to their distance to the nearest cellular membrane (gray). To elucidate whether the events of close proximity between fibrils and membranes were caused by chance or mediated by molecular interactions, random shifts (by 10 – 20 nm) and rotations (between 0 and 10°) were performed to the experimentally determined location of the fibrils. Black lines show 5 simulations for 50 randomly chosen fibrils. **e**, Measurements of inter-membrane distances for a 2D slice of the tomogram shown in **d**. The number of tomograms and biologically independent cryo-ET experiments is listed in Supplementary Table 1. Representative images are shown.

Supplementary Methods | Fibril-membrane and inter-membrane distance calculation.

Fibril-membrane distance

The algorithm computing fibril-membrane nearest distances can be summarized as follows:

For each tomogram:

1. Use the segmentation of organelle lumina to compute the distance transform tomogram[1], which calculates the Euclidean distance from each background voxel to the nearest segmented one.
2. For each fibril:

2.1. The curve defined by Amira's Xtracing module during segmentation is sampled uniformly each 5 nm (i.e. similar to the fibril radius).

2.2. For each point in the fibril:

2.2.1. To achieve subvoxel precision, get the interpolated value of the distance transform tomogram at the coordinates of that point.

2.2.2. Add this value to a list of fibril-membrane nearest distances.

The probability density was computed as the normalized histogram of the list of fibril-membrane nearest distances.

To test whether these fibril-membrane nearest distances resulted from random or specific interactions, we compared the experimentally determined distances with those of simulated fibrils. These simulated fibrils were created by randomly shifting and rotating the experimentally measured fibrils as follows:

For each tomogram, generate 200 synthetic tomograms:

1. Take randomly an input experimental fibril as reference.
2. Shift the reference fibril in respect to its center at a random distance in a range of [10, 20] nm.
3. Rotate the fibril randomly with respect to the fibril center with Euler angles selected randomly in the range of [0, 10] degrees.
4. Try to insert the resulting fibril in the synthetic tomogram. The insertion fails in the following cases:

4.1. The fibril intersects with another one, considering that fibrils have a cross-section radius of 5 nm.

4.2. The fibril intersects with a segmented membrane.

4.3. Part of the fibril is out of the tomogram boundaries.

5. Iterate until 50 fibrils are inserted or 5000 tries are reached.

Inter-membrane distance

The algorithm for computing inter-membrane nearest distances can be summarized as follows:

For each tomogram:

1. Assign labels for the lumen of each organelle.
2. Associate segmented membranes and lumina by a proximity criterion. For each voxel in a membrane segmentation, the label of the nearest lumen voxel is determined. The lumen is then associated to the membrane segmentation most frequently found.
3. For each lumen:

3.1. Compute the distance transform tomogram[1] from all lumina.

3.2. Erase the current lumen.

3.3. For each pixel on the membrane segmentation associated to the current lumen:

3.3.1. Get the interpolated value of the distance transform tomogram at the coordinates of that point.

3.3.2. Add this value to a list of inter-membrane nearest distances.

Probability densities were computed as described for fibril-membrane nearest distances.

References

1 van der Walt, S., Colbert, S. C. & Varoquaux, G. The NumPy Array: A Structure for Efficient Numerical Computation. *Comput Sci Eng* **13**, 22-30, doi:10.1109/mcse.2011.37 (2011).
