# Supplementary material for: In situ architecture of neuronal α-Synuclein inclusions
Source: Nat Commun. 2021 Apr 14;12:2110. doi: 10.1038/s41467-021-22108-0 (PMC8046968; doi:10.1038/s41467-021-22108-0)
Supplement: Supplementary file 3 — Description of Additional Supplementary Files [file 41467_2021_22108_MOESM3_ESM.docx]

**Description of Additional Supplementary Files**

File Name: Supplementary Movie 1| Tomogram and 3D rendering of an α-Syn aggregate in a neuron seeded by PFFs.

Description: Tomographic volume and 3D rendering of an inclusion seeded by PFFs in a neuron expressing GFP-α-Syn, shown in Fig. 1a, d. The field of view is 1.6 x 1.6 μm. The rendering depicts α-Syn fibrils (red), an autophagosome (cyan), ER (yellow), mitochondria (green) and various vesicles (purple).
