# Supplementary material for: In situ architecture of neuronal α-Synuclein inclusions
Source: Nat Commun. 2021 Apr 14;12:2110. doi: 10.1038/s41467-021-22108-0 (PMC8046968; doi:10.1038/s41467-021-22108-0)
Supplement: Supplementary file 5 — Reporting Summary [file 41467_2021_22108_MOESM5_ESM.pdf]

nature research

Corresponding author(s): F. Ulrich Hartl
Ruben Fernandez-Busnadiego

Last updated by author(s): Jan 9, 2021

Reporting Summary

Nature Research wishes to improve the reproducibility of the work that we publish. This form provides structure for consistency and transparency in reporting. For further information on Nature Research policies, see our Editorial Policies and the Editorial Policy Checklist.

Statistics

For all statistical analyses, confirm that the following items are present in the figure legend, table legend, main text, or Methods section.

n/a	Confirmed	
☐	☒	The exact sample size (n) for each experimental group/condition, given as a discrete number and unit of measurement
☐	☒	A statement on whether measurements were taken from distinct samples or whether the same sample was measured repeatedly
☐	☒	The statistical test(s) used AND whether they are one- or two-sided<br>*Only common tests should be described solely by name; describe more complex techniques in the Methods section.*
☒	☐	A description of all covariates tested
☒	☐	A description of any assumptions or corrections, such as tests of normality and adjustment for multiple comparisons
☐	☒	A full description of the statistical parameters including central tendency (e.g. means) or other basic estimates (e.g. regression coefficient) AND variation (e.g. standard deviation) or associated estimates of uncertainty (e.g. confidence intervals)
☐	☒	For null hypothesis testing, the test statistic (e.g. F, t, r) with confidence intervals, effect sizes, degrees of freedom and P value noted<br>*Give P values as exact values whenever suitable.*
☒	☐	For Bayesian analysis, information on the choice of priors and Markov chain Monte Carlo settings
☒	☐	For hierarchical and complex designs, identification of the appropriate level for tests and full reporting of outcomes
☒	☐	Estimates of effect sizes (e.g. Cohen's d, Pearson's r), indicating how they were calculated

Our web collection on statistics for biologists contains articles on many of the points above.

Software and code

Policy information about availability of computer code

Data collection	SerialEM 3.7.0<br>MAPS 2.1<br>Las X 3.5.7.23225<br>CellSens 2.1<br>FACSDiva Version 6.1.3
Data analysis	MotionCor2 1.2.1<br>IMOD 4.9.0<br>tom_deconv (https://github.com/dtegunov/tom_deconv)<br>tomoSegMemTV (https://github.com/anmartinezs/pyseg_system/tree/master/code/tomosegmemtv)<br>Amira 6.2<br>Matlab 2017a<br>ImageJ 2.0.0<br>Prism 6<br>Origin 2019b<br>PySeg (https://github.com/anmartinezs/pyseg_system/tree/master/code/pyorg/scripts/filaments; https://doi.org/10.5281/zenodo.4429140)<br>Persistence Length measurement (https://github.com/FJBauerlein/Huntington; https://doi.org/10.5281/zenodo.4428865)

For manuscripts utilizing custom algorithms or software that are central to the research but not yet described in published literature, software must be made available to editors and reviewers. We strongly encourage code deposition in a community repository (e.g. GitHub). See the Nature Research guidelines for submitting code & software for further information.

Data

Policy information about availability of data

All manuscripts must include a data availability statement. This statement should provide the following information, where applicable:

- Accession codes, unique identifiers, or web links for publicly available datasets
- A list of figures that have associated raw data
- A description of any restrictions on data availability

Data availability
Source Data for Fig. 2d, e, Fig. 4b, c, Supplementary Fig. 1f, g, Supplementary Fig. 3c, d, Supplementary Fig. 4 and Supplementary Fig. 6 are available with the online version of this paper. The individual values for the average graphs shown in Fig. 2d, Fig. 4b, c and Supplementary Fig. 4 are available at the Edmond repository: https://edmond.mpdl.mpg.de/imeji/collection/rnVkI2lwG8loNXOi. The tomograms shown in Fig. 1 and Fig. 2 are available in EMPIAR through accession codes EMD-11401 (Fig. 1a; https://www.ebi.ac.uk/pdbe/entry/emdb/EMD-11401), EMD-11417 (Fig. 1e; https://www.ebi.ac.uk/pdbe/entry/emdb/EMD-11417) and EMD-11416 (Fig. 2a; https://www.ebi.ac.uk/pdbe/entry/emdb/EMD-11416). All other data are available from the corresponding authors upon reasonable request.

Field-specific reporting

Please select the one below that is the best fit for your research. If you are not sure, read the appropriate sections before making your selection.

☒ Life sciences ☐ Behavioural & social sciences ☐ Ecological, evolutionary & environmental sciences

For a reference copy of the document with all sections, see nature.com/documents/nr-reporting-summary-flat.pdf

Life sciences study design

All studies must disclose on these points even when the disclosure is negative.

Sample size	Sample sizes were selected based on previous experience to obtain statistical significance and reproducibility (see e.g. Table S1 in Collado et al., Dev Cell 2019)
Data exclusions	Tomograms with insufficient signal-to-noise-ratio were excluded for all conditions.
Replication	Replicates were conducted for all experiments quantified as described in the Figure legends and Supplementary Table 1
Randomization	Mouse embryos of both sexes were chosen randomly for neuronal cell cultures. The MSA patient was selected based on histopathological brain analyses (ramdomization did not apply). Experiments on SH-SY5Y cells were performed comparing different experimental treatments on the same cell line (ramdomization did not apply).
Blinding	No blinding was applied. All experiments were performed comparing various treatments on otherwise comparable samples, and as such it was necessary for the researchers to be aware of the treatment applied (e.g. PBS, PFFs or MSA aggregates). Appropriate controls were included in each experimental replication.

Reporting for specific materials, systems and methods

We require information from authors about some types of materials, experimental systems and methods used in many studies. Here, indicate whether each material, system or method listed is relevant to your study. If you are not sure if a list item applies to your research, read the appropriate section before selecting a response.

Materials & experimental systems

n/a	Involved in the study	
☐	☒	Antibodies
☐	☒	Eukaryotic cell lines
☒	☐	Palaeontology and archaeology
☐	☒	Animals and other organisms
☐	☒	Human research participants
☒	☐	Clinical data
☒	☐	Dual use research of concern

Methods

n/a	Involved in the study	
☒	☐	ChIP-seq
☐	☒	Flow cytometry
☒	☐	MRI-based neuroimaging

Antibodies

Antibodies used	The following primary antibodies were used: GFP (A10262, Thermo Fisher, 1:500; RRID: AB_2534023), K48-linked ubiquitin (05-1307, Millipore; 1:500; RRID: AB_1587578), MAP2 (NB300-213, Novus Biologicals; 1:500; RRID: AB_2138178), p62 (ab56416, Abcam; 1:200; RRID: AB_945626), phospho S129 α-Syn (ab51253, Abcam; 1:500 for immunofluorescence, 1:2500 for western blot; RRID:

AB_869973), α-Syn (610787, BD Biosciences; 1:1000; RRID: AB_398108) and p62 lck ligand (610832, BD Biosciences; 1:100; RRID: AB_398151).
The following secondary antibodies were used: Alexa Fluor 488 AffiniPure Donkey Anti-Chicken (703-545-155, Jackson ImmunoResearch; 1:250), Alexa Fluor 647 AffiniPure Donkey Anti-Chicken (703-605-155, Jackson ImmunoResearch; 1:250), Cy3 AffiniPure Donkey Anti-Rabbit (711-165-152, Jackson ImmunoResearch; 1:250), Alexa Fluor 488 AffiniPure Donkey Anti-Mouse (715-545-150, Jackson ImmunoResearch; 1:250), Cy3-conjugated AffiniPure Goat Anti-Mouse IgG (115-165-003, Jackson ImmunoResearch; 1:1000), Cy3-conjugated AffiniPure Goat anti-rabbit (111-165-045, Dianova; 1:1000; RRID: AB_2338003), HRP-conjugated Goat Anti-Rabbit (A9169, Sigma; 1:5000; RRID: AB_258434).

Validation

All antibodies have been previously used for the application and species described here. The GFP Antibody has been validated for ICC in mice (https://www.thermofisher.com/antibody/product/GFP-Antibody-Polyclonal/A10262). The K48-linked ubiquitin Antibody has been validated for IF in human cell culture (https://www.merckmillipore.com/DE/de/product/Anti-Ubiquitin-Antibody-Lys48-Specific-clone-Apu2-rabbit-monoclonal,MM_NF-05-1307). The MAP2 antibody has been validated for IF in mouse primary cultures (https://www.novusbio.com/products/map2-antibody_nb300-213). The p62 antibody has been validated for IF in human cell culture (https://www.abcam.com/sqstm1--p62-antibody-autophagosome-marker-ab56416.html). The phospho S129 α-Syn antibody has been validated for WB with mouse brain lysates and has been used for IF in human cell culture (https://www.abcam.com/alpha-synuclein-phospho-s129-antibody-ep1536y-ab51253.html ; https://europepmc.org/backend/ptpmcrender.fcgi?accid=PMC7212628&blobtype=pdf). The α-Syn antibody has been validated for IHC in humans (https://www.bdbiosciences.com/eu/applications/research/stem-cell-research/ectoderm-markers/human/purified-mouse-anti--synuclein-42-synuclein/p/610787). The p62 lck ligand antibody has been validated for IHC in humans (https://www.bdbiosciences.com/us/reagents/research/antibodies-buffers/cell-biology-reagents/cell-biology-antibodies/purified-mouse-anti-p62-ick-ligand-3p62-lck-ligand/p/610832).

Eukaryotic cell lines

Policy information about cell lines

Cell line source(s)	SH-SY5Y cells were a gift from Konstanze Winklhofer and Joerg Tazelt (10.1038/emboj.2011.86) and orginially purchased at DSMZ (ACC209)<br>HEK293T cells were purchased from Takara (632180)
Authentication	No authentication was performed
Mycoplasma contamination	No contamination was found by PCR, electron and light microscopy.
Commonly misidentified lines<br>(See ICLAC register)	None

Animals and other organisms

Policy information about studies involving animals; ARRIVE guidelines recommended for reporting animal research

Laboratory animals	Primary cortical neurons were prepared from E15.5 CD-1 wild type mouse embryos of both sexes (breeding line MpiCrllcr:CD-1). Mice were housed in a specific pathogen free facility at 22 ± 1,5 °C, 55 ± 5% humidity, 14-hour light / 10-hour dark cycle.
Wild animals	no
Field-collected samples	no
Ethics oversight	All experiments involving mice were performed in accordance with the relevant guidelines and regulations of the Government of Upper Bavaria (Germany).

Note that full information on the approval of the study protocol must also be provided in the manuscript.

Human research participants

Policy information about studies involving human research participants

Population characteristics	A brain sample was collected from a male patient who died at the age of 54, 6 years after being diagnosed with a cerebellar type of MSA
Recruitment	The patient donated brain tissue to the Neurobiobank Munich. This sample was selected due to the abundant alpha-synuclein inclusions in the pons region
Ethics oversight	MSA patient brain tissue was obtained from Neurobiobank Munich. All autopsy cases of the Neurobiobank Munich are collected on the basis of an informed consent according to the guidelines of the ethics commission of the Ludwig-Maximilians-University Munich, Germany. The experiments performed in this paper were approved by the Max Planck Society's Ethics Council.

Note that full information on the approval of the study protocol must also be provided in the manuscript.

Flow Cytometry

Plots

Confirm that:

☒ The axis labels state the marker and fluorochrome used (e.g. CD4-FITC).

☒ The axis scales are clearly visible. Include numbers along axes only for bottom left plot of group (a 'group' is an analysis of identical markers).

☒ All plots are contour plots with outliers or pseudocolor plots.

☒ A numerical value for number of cells or percentage (with statistics) is provided.

Methodology

Sample preparation	To create a stable cell line expressing EGFP-α-SynA53T, SH-SY5Y cells were transfected using Lipofectamine 2000 (Thermo Fisher). Cells were cultured in in Dulbecco's modified Eagle's medium (DMEM, Biochrom) supplemented with 10 % fetal bovine serum (FBS, GIBCO), 2 mM L-glutamine (GIBCO) and 2,000 µg/ml geneticin for selection. Polyclonal cell lines were generated by fluorescence-activated cell sorting. Upon selection, cells were cultured in medium supplemented with 200 µg/ml geneticin (Thermo Fisher) and penicillin/streptomycin (Thermo Fisher).<br><br>Negative control for FACS: untransfected SH-SY5Y cells
Instrument	BD FACS Aria III with 375nm, 405nm, 488nm, 561nm, 633nm lasers
Software	FACSDiva Version 6.1.3
Cell population abundance	All sorted cells showed GFP signal
Gating strategy	Control cells (untransfected SH-SY5Y cells): FITC-A: 120 FSC-A: 150.000<br>Transfected SH-SY5Y cells: FITC-A: 10.000 FSC-A: 150.000

☐ Tick this box to confirm that a figure exemplifying the gating strategy is provided in the Supplementary Information.
